# Comparative Analyses of Complete Chloroplast Genomes and Karyotypes of Allotetraploid *Iris koreana* and Its Putative Diploid Parental Species (*Iris* Series *Chinenses*, Iridaceae)

**DOI:** 10.3390/ijms231810929

**Published:** 2022-09-18

**Authors:** Inkyu Park, Bokyung Choi, Hanna Weiss-Schneeweiss, Soonku So, Hyeon-Ho Myeong, Tae-Soo Jang

**Affiliations:** 1 Department of Biological Science, College of Bioscience and Biotechnology, Chungnam National University, Daejeon 34134, Korea; 2 Department of Biology and Chemistry, Changwon National University, Changwon 51140, Korea; 3 Department of Botany and Biodiversity Research, University of Vienna, Rennweg 14, A-1030 Vienna, Austria; 4 Plant Conservation Center, Korea National Park Research Institute, 2 Baengnyeonsa-gil, Seolcheon-Myeon, Muju-gun 55557, Korea; 5 Korea National Park Research Institute, 171 Dangu-ro, Wonju-si 26441, Korea

**Keywords:** allopolyploidy, *Iris* series *Chinenses*, FISH, rDNA loci, chloroplast genome

## Abstract

The *Iris* series *Chinenses* in Korea comprises four species (*I. minutoaurea*, *I. odaesanensis*, *I. koreana,* and *I. rossii*), and the group includes some endangered species, owing to their high ornamental, economic, and conservation values. Among them, the putative allotetraploid, *Iris koreana* (2*n* = 4*x* = 50), is hypothesized to have originated from the hybridization of the diploids *I. minutoaurea* (2*n* = 2*x* = 22) and *I. odaesanensis* (2*n* = 2*x* = 28) based on morphological characters, chromosome numbers, and genome size additivity. Despite extensive morphological and molecular phylogenetical studies on the genus *Iris*, little is known about Korean irises in terms of their complete chloroplast (cp) genomes and molecular cytogenetics that involve rDNA loci evolution based on fluorescence in situ hybridization (FISH). This study reports comparative analyses of the karyotypes of the three *Iris* species (*I. koreana*, *I. odaesanensis*, and *I. minutoaurea*), with an emphasis on the 5S and 35S rDNA loci number and localization using FISH together with the genome size and chromosome number. Moreover, the cp genomes of the same individuals were sequenced and assembled for comparative analysis. The rDNA loci numbers, which were localized consistently at the same position in all species, and the chromosome numbers and genome size values of tetraploid *Iris koreana* (four 5S and 35S loci; 2*n* = 50; 1C = 7.35 pg) were additively compared to its putative diploid progenitors, *I. minutoaurea* (two 5S and 35S loci; 2*n* = 22; 1C = 3.71 pg) and *I. odaesanensis* (two 5S and 35S loci; 2*n* = 28; 1C = 3.68 pg). The chloroplast genomes were 152,259–155,145 bp in length, and exhibited a conserved quadripartite structure. The *Iris* cp genomes were highly conserved and similar to other Iridaceae cp genomes. Nucleotide diversity analysis indicated that all three species had similar levels of genetic variation, but the cp genomes of *I. koreana* and *I. minutoaurea* were more similar to each other than to *I. odaesanensis*. Positive selection was inferred for *psbK* and *ycf2* genes of the three *Iris* species. Phylogenetic analyses consistently recovered *I. odaesanensis* as a sister to a clade containing *I. koreana* and *I. minutoaurea*. Although the phylogenetic relationship, rDNA loci number, and localization, together with the genome size and chromosome number of the three species, allowed for the inference of *I. minutoaurea* as a putative maternal taxon and *I. odaesanensis* as a paternal taxon, further analyses involving species-specific molecular cytogenetic markers and genomic in situ hybridization are required to interpret the mechanisms involved in the origin of the chromosomal variation in *Iris* series *Chinenses*. This study contributes towards the genomic and chromosomal evolution of the genus *Iris*.

## 1. Introduction

Iridaceae is considered a monophyletic family and is one of the ornamentally most important groups of angiosperms [1,2,3,4]. The genus *Iris* L. is known to be morphologically diverse and comprises approximately 14 species in Korea [5]. *Iris* section *Liminris* series *Chinenses* comprises seven species worldwide, of which, four species are distributed in Korea [6]. Among the four species, *I. rossii* Baker (2*n* = 32) and *I. minutoaurea* Makino (2*n* = 22) are relatively widely distributed in Korea, and their distribution extends to China and Japan. The other two *Iris* species, *I. odaesanensis* Y.N.Lee (2*n* = 28) and *I. koreana* Nakai (2*n* = 50), have a restricted geographical distribution [7,8]. *Iris koreana* is endemic to Korea and *I. odaesanensis* is subendemic to Korea, with a disjunct population in Jilin Province, China [7,8]. The remaining three taxa, which were newly described or taxonomically recently revised, are endemic to China, and their chromosome numbers have not been reported to date [6]. Two of the diploid species (*I. minutoaurea* and *I. odaesanensis*) were hypothesized to be involved in the formation of the allotetraploid (*I. koreana*) via a single allopolyploidization event [8]. Among the four *Iris* series *Chinenses* species in Korea, *I. rossii* differs from the other three Korean species in terms of floral [5,9], pollen [10], and leaf indumentum [11] morphologies. In phylogenetic analyses based on the whole-chloroplast genome or nuclear internal transcribed spacer (ITS) region, *Iris rossii* is a sister to a clade containing *I. odaesanensis*, *I. minutoaurea,* and *I. koreana* [12]. In contrast, the three remaining species, *I. odaesanensis*, *I. minutoaurea,* and *I. koreana*, share similar morphological traits (e.g., leaf width, tepal shape, and size) but differed in some floral morphological characters (e.g., tepal color and floral tube length) [5,9]. The three irises were shown to be a monophyletic group based on molecular phylogenetic analyses using complete chloroplast (cp) genomes, chloroplast (*mat*K), and nuclear DNA sequences (ITS) [7,12,13,14].

To date, despite numerous evolutionary studies based on pollen and leaf micromorphology [10,11], whole chloroplast genome sequences [12,15], and molecular phylogenetic [7,13,14] and cytogenetic analyses [8,16] in angiosperms, understanding the diversification and speciation processes following polyploidization in wild plant groups is challenging. The chromosome number and karyotype structural changes accompanying hybridization and polyploidization play crucial roles in plant evolution [17,18]. Karyotype analysis has long been used as a source of basic genomic information [8,18,19,20,21]. The advent of molecular cytogenetics, particularly fluorescence in situ hybridization (FISH) methods, has allowed for more in-depth analyses of karyotypes. However, analyses of chromosome complements and their evolution in most non-model plant groups have been hampered by the lack of informative chromosomal marker sequences [22,23,24,25]. The molecular cytogenetic mapping of nuclear ribosomal RNA genes (5S and 35S rDNAs) has often provided insights into karyotype characterization, including the number and localization of rDNA as well as the evolution of flowering plant groups [26,27,28,29,30]. The 35S rDNA loci comprising 18S-5.8S-25S rDNA are located in the nucleolar organizer regions, whereas tandem arrays of 5S rDNA loci are generally found independently on chromosomes [18]. Thus, the molecular cytogenetic mapping of rDNA loci using FISH in closely related species frequently allows for conclusions toward a better understanding of the chromosomal rearrangements in taxonomically complicated groups of plants [19,24]. Despite a growing wealth of data on the karyotype structure of Iridaceae taxa [28,31,32,33], such information is still largely lacking for Korean irises and thus needed for a better understanding of their chromosomal evolution.

Chloroplasts are essential organelles that are involved in photosynthesis and play important roles in plant carbon fixation, the biosynthesis of starch, fatty acids, amino acids, pigments, and energy transformation [34,35,36]. The assembled cp genomes of angiosperms range from 72 to 217 kb in size, and they typically exhibit a quadripartite structure with two copies of inverted repeats (IRs) separated by a large single copy (LSC) and small single copy (SSC) regions [37,38,39]. Most cp genomes of angiosperms contain 110–130 genes, with up to 80 protein-coding genes and transcription and translation-related genes, 30 transfer RNA (tRNA), and 4 ribosomal RNA (rRNA) genes [34,35]. Although cp genomes have a highly conserved structure, gene content, and typically low levels of DNA sequence variation compared to nuclear and mitochondrial genomes, whole chloroplast genomes have been used in reconstructing the phylogenetic relationships among closely related plant species [40,41,42,43,44]. Several studies have reported the complete cpDNA genome analyses of Iridaceae species [45,46,47,48]; however, so far, only a few cp genomes of Korean *Iris* species have been sequenced and analyzed using phylogenomic approaches [12,15]. Despite their extraordinary economic importance as medicinal and ornamental plants [1,2,3,4,49,50,51], data on karyotypic characters of Korean irises are limited, and this hampers the analyses of their origin and the further evolution of polyploid species.

*Iris koreana* (2*n* = 50; 1C = 7.29 pg) has been hypothesized to have originated via allopolyploidization between *I. minutoaurea* (2*n* = 22; 1C = 3.85 pg) and *I. odaesanensis* (2*n* = 28, 3.72 pg/1C) [7,8,10,11]. Among the three species, *I. koreana* and *I. odaesanensis* are of major conservation concern due to rapid decreases in their population sizes [5,52,53]. Despite this, genomic resources of the ser. *Chinenses* are still relatively scarce. Some progress has been made by sequencing cp genomes of Korean irises [12,15]. However, understanding the evolution of hybrid or allopolyploid taxa using molecular data alone is still a major challenge and the chloroplast genome of the group has not been comparatively analyzed together with molecular cytogenetic data [54,55]. Thus, a combination of genomic and molecular cytogenetic approaches is needed to gain better insights into the evolution and relationships of the three closely related *Iris* species [56,57].

This study aims to analyze the relationships and evolutionary trajectories of the three closely related species of *Iris* series *Chinenses*, that is, the two diploids (*I. minutoaurea* and *I. odaesanensis*) and one tetraploid (*I. koreana*) species, using molecular cytogenetic analyses of their karyotypes and cp genome sequence analysis. The specific aims of the present study are to (1) analyze the karyotypes of two diploids and a tetraploid species for the first time, with a special emphasis on 5S and 35S rDNA loci evolution, (2) comparatively analyze de novo-assembled complete cp genome sequences of the three closely related irises, and (3) analyze the chloroplast genome evolution of the diploids and their progenitor tetraploid within the phylogenetic framework.

## 2. Results

**Figure 1:**
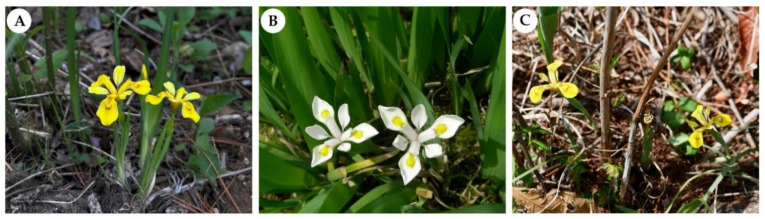
Morphology and habit of the three *Iris* species from Korea. (**A**) *I. koreana*, (**B**) *I. odaesanensis*, (**C**) *I. minutoaurea*.

The three species possessed similar morphological traits (e.g., leaf width, tepal shape, and size) but differed in some floral morphological characters (e.g., tepal color and floral tube length). *Iris odaesanensis* differed from *I. koreana* and *I. minutoaurea* in having white tepals rather than typically yellow tepals (Figure 1A–C). *I. minutoaurea* was characterized by an unbranched flowering stem with a solitary flower (Figure 1C), whereas two-branched flowering stems were mainly observed in *I. koreana* (Figure 1A).

### 2.1. Localization and Number of rDNA Loci in Iris koreana, I. minutoaurea, and I. odaesanensis

**Figure 2:**
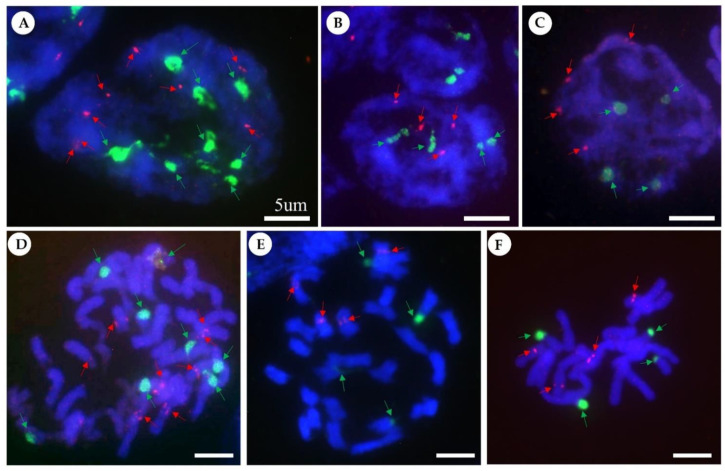
Localization of 5S and 18S rDNA loci in nuclei (**A**–**C**) and metaphase (**D**–**F**) of chromosomes of the three *Iris* species. Fluorescence in situ hybridization (FISH) was performed with 5S rDNA (red signals) and 18S rDNA (green signals) as probes. (**A**,**D**) *Iris koreana*, (**B**,**E**) *I. odaesanensis*, and (**C**,**F**) *I. minutoaurea*. Scale bar = 5 µm.

The number and localization of rDNA loci were determined using 5S and 18S rDNA probes for FISH. The numbers of rDNA loci of the three *Iris* species are reported here for the first time (Table 1, Figure 2). 18S rDNA loci were all located in the subterminal regions of the short arms (Figure 2D–F). All 5S rDNA loci were localized in pericentric regions of chromosomes in all investigated taxa (Figure 2D–F). Both *I. minutoaurea* and *I. odaesanensis* had two of each 5S rDNA and 18S rDNA loci (Figure 2B,C,E,F), whereas *I. koreana* chromosomes had four each (Figure 2A,D). Chromosome numbers and genome sizes, as well as the numbers and distribution patterns of 5S and 18S rDNA loci of the allotetraploid species, *Iris koreana* (2*n* = 4*x* = 50; 1C = 7.35 pg), were additive compared to its putative diploid progenitors *I. minutoaurea* (2*n* = 2*x* = 22; 1C = 3.71 pg) and *I. odaesanensis* (2*n* = 2*x* = 28; 1C = 3.68 pg) (Figure 2, Table 1).

### 2.2. Organization of the Iris Chloroplast Genome

Chloroplast genomes of *I. koreana*, *I. minutoaurea*, and *I. odaesanensis* were sequenced at approximately 502×, 556×, and 687× coverage, respectively, generating 3.2–3.4 Gb of raw paired-end read data and 2.7–2.9 Gb of trimmed reads (Figure S1, Tables S1 and S2).

**Figure 3:**
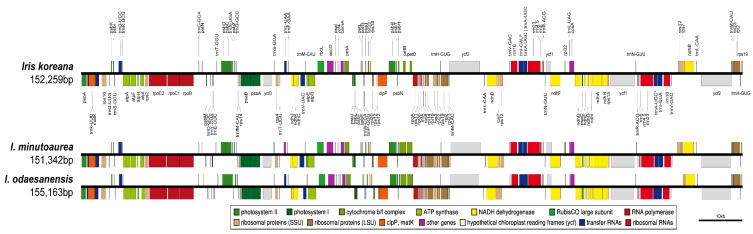
Linear gene map of the chloroplast genomes of the three analyzed *Iris* species. Genes are transcribed from the left to right. Genes indicated above the first black horizontal line from the top are positioned in the forward direction (left to right). Genes right below the first black horizontal line are positioned in the reverse direction (left to right).

The cp genomes of *Iris* species exhibited a typical quadripartite structure (Figure 3 and Figure S2). The complete cp genomes of the three *Iris* species varied from 151,342 bp (*I. minutoaurea*) to 155,163 bp (*I. odaesanensis*) in length. LSC regions ranged from 81,900 bp to 83,879 bp, SSC regions from 18,358 bp to 18,722 bp, and IR regions from 25,542 bp to 26,281 bp in length, and the three regions were the shortest in *I. minutoaurea* and the longest in *I. odaesanensis* (Table 2). The total guanine and cytosine (GC) content (37.8%) was consistent in the three *Iris* cp genomes. In general, the GC content of the IRs (43.1–43.2%) was higher than those of the LSC (36.0%) and SSC (31.1–31.2%) regions (Table 2). All cp genomes consistently had 114 genes, and the genes included 79 protein-coding, 4 rRNA and 31 tRNA genes (Table 2 and Table S3). The cp genomes contained 18 intron-containing genes, 16 of which had a single intron and 2 of which (*ycf3* and *clpP*) had two introns with duplicate genes (*ndhB*, *trnI-GAU*, and *trnA-UGC*) within the IR regions (Table S4).

### 2.3. Repetitive DNA Sequences in Iris Chloroplast Genome

**Figure 4:**
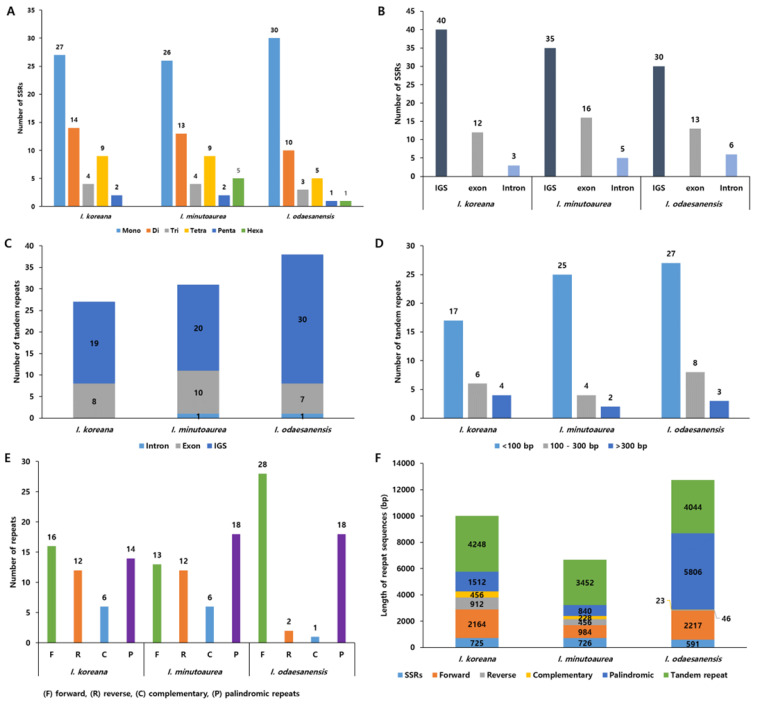
Distribution of the repeat sequences in *Iris* cp genomes. (**A**) Distribution of SSR motif types (mono-, di-, tri-, tetra-, penta-, and hexanucleotides). (**B**) Distribution of simple sequence repeats (SSRs) in intergenic spacers (IGS), exons, and introns. (**C**) Number and distribution of tandem repeats. (**D**) Abundance of tandem repeats sorted by length. (**E**) Abundance of forward (**F**), reverse (R), complementary (**C**), and palindromic (P) repeats. (**F**) Profiles of the repeat length distribution in cp genomes and total length of repeats (in bp).

The presence of simple sequence repeats (SSRs), tandem, forward, reverse, complementary, and palindromic repeats was tested to identify repetitive DNA sequence types in the cp genomes of the three *Iris* species. The *Iris* cp genomes contained repeat sequences within the intergenic spacer (IGS) regions. In total, 50 SSRs were identified in *I. odaesanensis,* and 59 SSRs in *I. minutoaurea* (Figure 4A). The mononucleotide motifs were the most abundant in all cp genomes studied, followed by dinucleotides (Figure 4A). SSRs were predominantly found in IGS regions (Figure 4B). The *Iris* cp genomes possessed numerous tandem repeats located in IGS regions and were typically less than 100 bp long, and only 2–4 tandem repeats >300 bp in length were identified in all three accessions (Figure 4C,D). The number of repeats in the *Iris* cp genomes were 13–28 forward, 2–12 reverse, 1–6 complementary, and 14–18 palindromic repeats, respectively (Figure 4E). Overall, most repeats (measured by total length) found in *I. koreana* and *I. minutoaurea* were represented by tandem and forward repeats, whereas *I. odaesanensis* possessed predominantly palindromic and tandem repeats (Figure 4F).

### 2.4. Comparative Analysis of Iris Chloroplast Genomes

**Figure 5:**
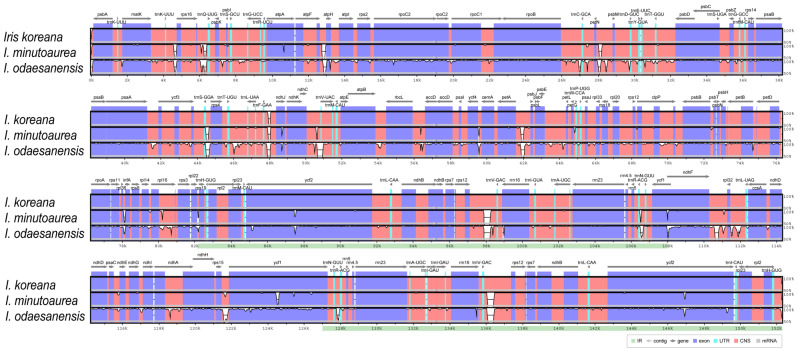
Visualization of the comparison of the three *Iris* cp genomes using mVISTA, with *I. koreana* as the reference. Different colors represent different coding and non-coding gene regions. Dark-blue blocks: conserved genes, light-blue blocks: transfer RNAs (tRNAs) and ribosomal RNAs (rRNAs), red blocks: conserved non-coding sequences (CNS). The vertical scale with white represents the regions with higher levels of sequence variations within the chloroplast genome.

**Figure 6:**
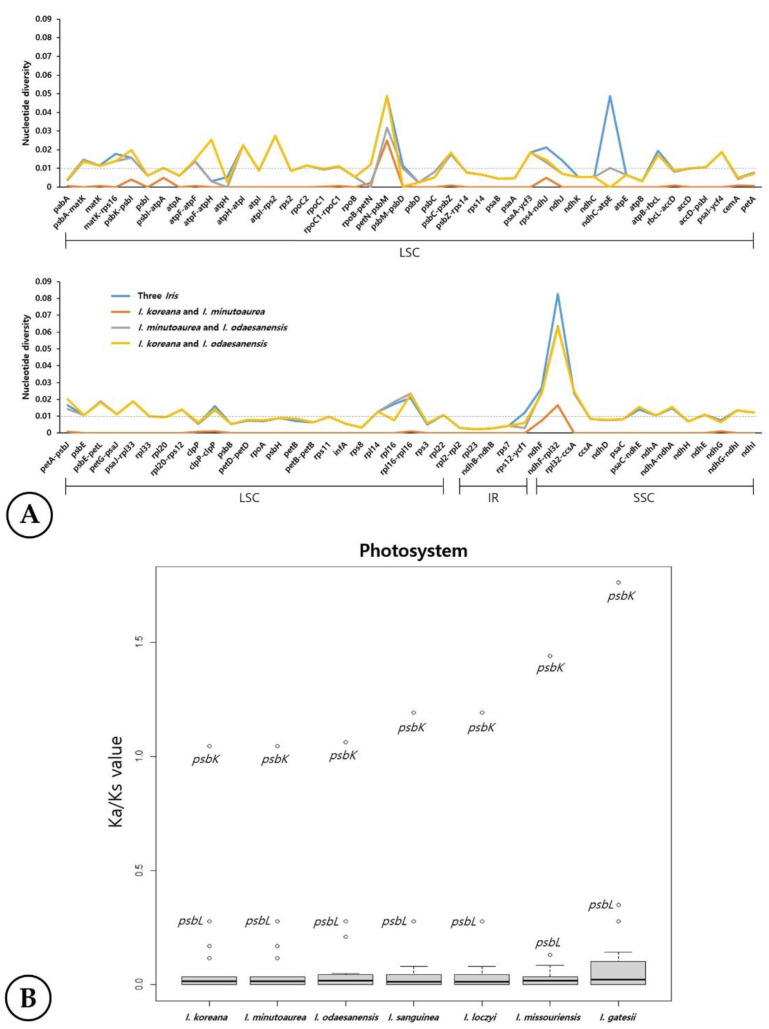
(**A**) Comparisons of the nucleotide diversity (Pi) values among the cp genes of the three analyzed *Iris* species. The mean of Pi value for all analyzed genes of the three *Iris* species is indicated with the dotted line. (**B**) Ka/Ks values for the photosystem-related genes in the *Iris* cp genomes.

The sequence identities were analyzed with mVISTA software using the *I. koreana* cp genome as a reference because it is a putative allotetraploid between diploid *I. minutoaurea* and *I. odaesanensis* (Figure 3). Overall, the *Iris* cp genome structure was well conserved among the three species (Figure 5), with the genic regions being more conserved than the IGS regions. The highest divergences were observed in the LSC and SSC regions in all three species (Figure 6). The average nucleotide diversity (Pi) for the three *Iris* cp genomes was calculated as 0.012. The three *Iris* cp genomes were divergent (hotspots regions) in the non-coding *petN-psbM* and *ndhC-atpE* in the LSC region, as well as in *ndhF-rpl32* within the SSC region (Figure 6). Furthermore, *ndhF-trnL* exhibited the highest Pi value of 0.0826, followed by *ndhC-atpE* (0.0489) within the SSC region (Figure 6). Although all three species had similar patterns of Pi values in the analyzed genes, *I. koreana* and *I. minutoaurea* cp genomes were more conserved when the two species were compared against each other than *I. minutoaurea* and/or when *I. koreana* was compared against *I. odaesanensis*. The IRs were more conserved than in single-copy regions. The comparison of the syntenic regions and sequence identities among the three *Iris* species (Figure S3) revealed that the cp genomes of the *Iris* had highly conserved collinear blocks, and, thus, the overall genome structure and gene order were variable mostly within some particular regions (Figure 5).

Comparison of IR contraction and expansion in the *Iris* species revealed overall similar IR lengths (ranging from 25,217 to 26,328 bp), with some differences in IR expansions and contractions (Figure S4). The *rpl22* gene was located entirely in the LSC region (Figure S4). The *ycf1* genes were located within the IRb/SSC and SSC/IRa junctions. Overall, the IRs were found to have experienced expansion in all cp genomes analyzed. The *pabA* gene was located in the LSC region, 63–174 bp from the IRa/LSC boundary. The *rps19* genes were duplicated in the IR regions flanking the border junctions.

### 2.5. Selective Pressure in Iris cp Genomes

Analyses of the non-synonymous substitution and synonymous-substitution (Ka/Ks) ratios using the *Crocus sativus* cp genome as a reference allowed for the identification of 78 genes that showed evidence of selective pressure in the seven *Iris* cp genomes (Figure S5). Most genes were conserved and exhibited relaxed selection (0 < Ka/Ks ratio < 1). No significant gene diversification was observed within LSC, IR, or SSC regions of cp genomes. The average Ka and Ks values were 0.019 and 0.135, respectively, with 64 genes having Ka and Ks values higher than 0.001 (Figure S5). The highest Ks value of 0.352 was inferred for *rpl33* gene in the cp genome of *I. gatesii*. Positive selection was observed in the *ycf2* gene in all but *I. missouriensis* and *I. gatesii* species (Figure 6 and Figure S5). The Ka/Ks ratios for most photosynthetic apparatus genes were close to 0. The highest Ka/Ks ratio of 1.762 was recovered for the *psbK* gene of *I. gatesii*. In all seven *Iris* cp genomes used for comparative analyses, the *psbK* genes showed evidence for positive selection (Ka/Ks ratio of 1.046–1.762; Figure 6). Other genes indicated low levels of variation (Figure S6). Thus, in general, positive selection pressures (Ka/Ks > 1) were observed for *psbK* and *ycf2* genes in the analyzed *Iris* species (Figure 6).

### 2.6. Phylogenic Relationships within the Iris Species based on Whole CP Genome Sequences

**Figure 7:**
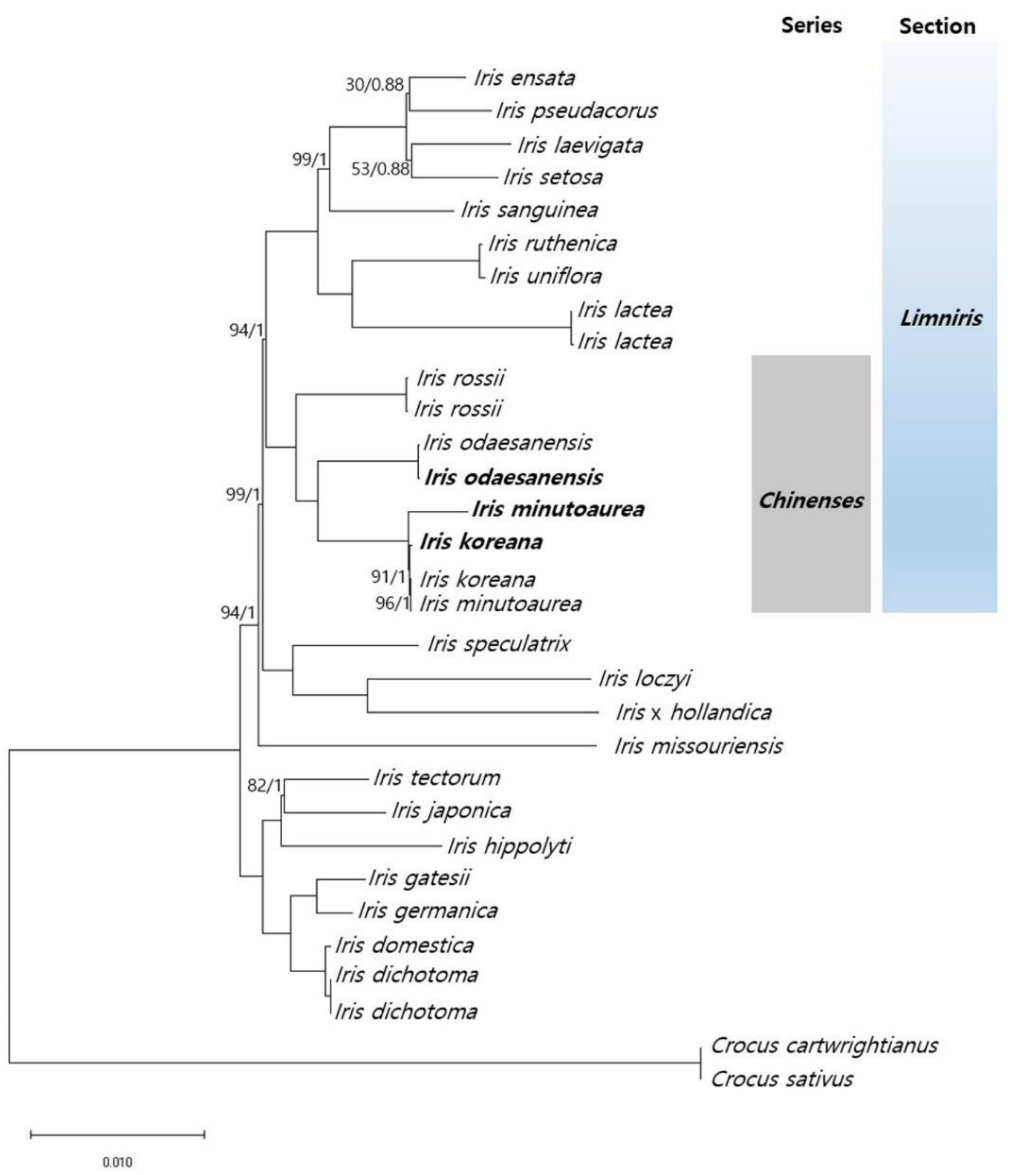
Phylogenic analyses of 29 *Iris* samples inferred from maximum likelihood (ML) and Bayesian inference (BI)-based analyses on 78 coding sequences. Branches with support of 100/1 were not indicated and maximum posterior probability (PP) are indicated above as the following (BS/PP). The newly included cp genomes by this study are indicated in bold.

Whole chloroplast genome sequences of *Iris* species available to date were used together with the cp genomes that were newly sequenced in this study to test phylogenetic relationships within the genus, with *Crocus cartwrightianus* and *C. sativus* serving as an outgroup (Figure 7). Maximum likelihood (ML) and Bayesian inference (BI) analyses using 79 protein-coding regions of cp genomes resulted in highly congruent topologies (Figure 7 and Figure S7). The tree topologies indicated strong support for the monophyly of *Iris* ser. *Chinenses* (BS = 100, posterior probability (PP) = 1.0; Figure 7). The phylogenetic relationships among most taxa were highly supported, suggesting that the cp genome data were significantly increased in resolution in a systematic context in Korean Iridaceae (Figure 7). Within the series, *I. rossii* was recovered as the sister species to a monophyletic clade containing three Korean irises, *I. odaesanensis* (2*n* = 2*x* = 28), *I. minutoaurea* (2*n* = 2*x* = 22), and *I. koreana* (2*n* = 4*x* = 50; Figure 7). Within the clade containing the three *Iris* species, *I. minutoaurea* formed a clade with *I. koreana*, and the two species were sisters to *I. odaesanensis.* The close phylogenetic relationship of *I. minutoaurea* and *I. koreana* supported the inference of *I. minutoaurea* as the maternal parent of the tetraploid species, *I. koreana*.

## 3. Discussion

The positions of other *Iris* species in the phylogeny were largely consistent with the previously published Iridaceae classification system [12,15,58,59,60,61]. Gross morphology and growth habit are often insufficient for clear species delimitation of the three Korean *Iris* species analyzed here [7,13,14,62]. Diploid *Iris odaesanensis* is morphologically distinguishable from the two other closely related species based on tepal color and tube length [5,6,9]. Diploid *I. minutoaurea* and tetraploid *I. koreana*, however, are very similar in morphology, especially in growth habit and tepal color, despite the fact that the former has an unbranched flowering stem whereas the latter has a branched flowering stems [5,9]. This morphological similarity of the latter two species led to a hypothesis that *I. koreana* is a putative allotetraploid species resulting from the hybridization of *I. odaesanensis* and *I. minutoaurea,* and this hypothesis is further supported by the additivity of two putative parental diploid species in the rDNA (5S and 18S) loci number and localization, chromosome numbers, and genome sizes corresponding to those in the tetraploid *I. koreana* [8].

### 3.1. Molecular Cytological Characterization

The present study provided the first report on the rDNA loci number and localization for Korean irises. Despite the differences in chromosome numbers among the three analyzed species, two diploid species exhibit the same number and location of their rDNA loci, whereas the tetraploid species is additive with respect to the rDNA loci numbers of the two putative parental diploid species with the same rDNA localization, which possibly supports its allopolyploid origin. The putative allotetraploid species *I. koreana* (2*n* = 50) has a genome size that is equal to the sum of the genome sizes of the parental diploid species, *I. minutoaurea* (2*n* = 22) and *I. odaesanensis* (2*n* = 28) [8]. The additivity of rDNA loci and the chromosome number may suggest a lack of gross genomic rearrangements in the polyploid and/or its recent origin [10,11,62]. In general, genome evolution in allopolyploids could be affected by diploidization or chromosomal rearrangements [63], or amplification of repetitive DNA, which is responsible for genome size variation as observed in plants [64]. This repetitive DNA is predominantly composed of dispersed repeats (e.g., DNA transposons and retroelements) and genus/species specific tandem satellite DNAs [18]. Thus, further studies employing genomic in situ hybridization (GISH; i.e., the mapping of genomic DNAs of the putative parental taxa to allopolyploid chromosomes [24]) analysis and the mapping of other species-specific satellite DNA repeats is required for a better understanding of genome evolution in the closely related group of species, as previously used for evolutionary analyses of other natural allopolyploid taxa [24,29,65,66,67].

### 3.2. Features of Iris Chloroplast Genomes

The organizations of the complete plastid (cp) genomes of the three *Iris* species reported here were similar to those of other *Iris* cp genomes [12,15,45,46,47,48]. All genomes exhibited a typical quadripartite structure, with LSC and SSC regions separated by IRs. The newly assembled *Iris* cp genomes carried 114 unique genes, and the gene order, GC content, and overall length (151,342–155,163 bp) were similar to published *Iris* cp genomes [12,15,45,46,47,48].

SSRs/microsatellites of one to six nucleotides motifs are widely distributed in most genomes [68]. SSRs are often used for genetic and population analyses and for species discrimination. They are also employed in phylogenetic studies because of their high levels of polymorphisms at both intra- and interspecific levels. In this study, approximately 66–80% of the mono- and dinucleotide SSRs (39–41 SSRs) were detected within the IGS regions. This finding is similar to those of previous reports, where most mononucleotide repeats were AT-rich due to an abundance of polyamines and polythymines in the cp genome [68,69,70]. The identification and characterization of SSRs in *Iris* cp genomes provide useful tools for species identification and population genetic analyses of *Iris* species. Tandem repeats are often actively involved in the changes in genome structure by promoting genome rearrangements [71]. Most tandem repeats detected in the *Iris* cp genomes were less than 100 bp in length but repeats with over 300 nucleotides were also found. Among all of the *Iris* species, *I. koreana* had the fewest tandemly repeated sequences in the cp genome.

### 3.3. Genetic Diversity of the Three Iris Chloroplast Genomes

Chloroplast genomes of *Iris* species exhibited low levels of diversity, and their genic regions were more conserved than that of intergenic spacer regions, which is in congruence with data from other angiosperm cp genomes [72,73,74]. Comparative analysis of the genetic variation at the species level using Pi values revealed that most divergent regions were non-coding, which is generally consistent with other *Iris* cp genomes [12] and other plant cp genomes in general. Chloroplast genomes of *Iris* species were highly variable in non-coding regions, especially spacers of *petN-psbM* and *ndhC-atpE* in the LSC region and of *ndhF-rpl32* in the SSC region. These regions were previously found to be hotspots of genetic variation [75,76,77]. Overall, the patterns of genetic variation were very similar in all three *Iris* species. However, cp genomes of *I. koreana* and *I. minutoaurea* were similar to each other and exhibited similar levels of variation compared to *I. odaesanensis,* which may additionally support that *I. minutoaurea* was likely involved in the hybridization with *I. odaesanensis* as the maternal parent to form allotetraploid *I. koreana*, in addition to the chromosome number, rDNA number, and localization, as well as C-value data.

IR regions of the cp genome are typically more conserved than single-copy regions. The variable regions identified in *Iris* cp genomes can be further used for DNA barcoding and thus species delimitation. The contraction and expansion of IRs in angiosperm cp genomes is often associated with cp genome size variation [78]. Previous studies have identified extremely short IRs, or have even reported the entire loss of IR regions and genes in plants [79,80]. Although structures of IRs and gene positions in *Iris* cp genomes are conserved, the length of the IR regions has been found to vary, ranging from 25,799 to 26,328 bp. This suggests a certain level of contraction/expansion of the IR regions during species evolution and diversification. The *Iris* cp genomes reported here had contracted IR, which agrees with previous reports of other *Iris* cp genomes [12,15,45,46,47,48].

The most conserved genes in *Iris* cp genomes exhibited a purifying selection. Both *psbK* and *ycf2*, which commonly experience selective pressure in cp genomes, also showed signs of positive selection in *Iris* cp genomes as commonly observed in angiosperm cp genomes [81,82,83,84,85]. Genes showing positive selection are considered to be involved in an adaptive evolution in response to extreme environmental changes, such as salt stress, cold temperatures, and high irradiation [81,82,83,84,85,86].

### 3.4. Phylogenetic Relationships in the Iridaceae

Comparative chloroplast genome data were widely used to construct a phylogenetic relationship in *Iris* species, and our ML and BI analyses consistently supported *Iris* series *Chinenses* as a monophyletic group [12,59]. Phylogenetic analyses recovered *I. odaesanensis* as the sister species to a clade encompassing *I. koreana* and *I. minutoaurea*, which agrees with previous studies [12], providing further evidence of diploid *I. minutoaurea* (2*n* = 22) as a maternal parental species of the tetraploid *I. koreana* (2*n =* 50). The paternal parental species was hypothesized to be the diploid *I. odaesanensis* (2*n* = 28). These phylogenetic data support an earlier hypothesis of the origin of *I. koreana* that was inferred from morphological similarities (leaf indumentum and pollen micromorphology), as well as the chromosome number and genome size additivity [8,10,11].

The current study presents comprehensive analyses of Korean *Iris* species of ser. *Chinenses*, focusing on analyses of two closely related diploids and one putative tetraploid species involved in hybridization. It presents new data that support the evolutionary relationships of the three closely related taxa, focusing on in-depth cytogenetic and phylogenetic analyses [12,15,45,46,47,48]. Further molecular cytogenetic analyses employing the GISH technique on the origin and evolution of *Iris* ser. *Chinenses,* which includes the newly described species of the series (specifically *I. dabashanensis*, *I. probstii*, and *I. speculatrix*), together with all of the Korean species in the group, are needed for a better understanding of the diversification and speciation of the genus *Iris*, including its polyploidization and hybridization [6].

## 4. Materials and Methods

### 4.1. Taxon Sampling

All plants analyzed in this study were collected from natural populations or sourced from cultivated material in Korea [8,10,11,15]. To minimize damage to plant populations, the same individuals (collection numbers JC041913 for *I. minutoaurea*; BKC928 for *I. odaesanensis*; sck00043 for *I. koreana*; [8]) were used for chloroplast genome sequencing and molecular cytogenetic analyses, as well as genome size information (Table 1; [8]).

### 4.2. Molecular Cytogenetic Analysis

Actively growing root meristems were pretreated with 0.05% aqueous solution of colchicine at room temperature for 4.5 h, fixed in ethanol:acetic acid (3:1) for at least 3 h, and stored at −20 °C until use. Fixed root meristems were prepared by enzymatic digestion and squashing as described in Jang and Weiss-Schneeweiss [87].

FISH was performed using the established protocol of Jang and Weiss-Schneeweiss [87]. Probes used for FISH were complete coding regions of 18S rDNA from *Arabidopsis thaliana* in plasmids pSK^+^ [88], and the genic region of 5S rDNA isolated from *Melampodium montanum* in plasmid pGEM-T Easy [19]. The probes were labelled either with biotin-16-dUTPs or digoxygenin-11-dUTPs (Roche, Vienna, Austria) directly using PCR (5S rDNA) or using a nick translation kit (18S rDNA; Roche, Vienna, Austria). Digoxigenin was detected with anti-digoxigenin conjugated with fluorescein isothiocyanate (5 µg/mL; Roche, Vienna, Austria) and biotin with ExtrAvidin conjugated with Cy3 (2.5 µg/mL; Sigma-Aldrich, Vienna, Austria). All FISH analysis and image capturing using AxioImager M2 epi-fluorescent microscope (Carl Zeiss, Vienna, Austria) were performed as described in the study by Jang et al. [24]. At least 10 well-spread mitotic metaphases and prometaphases were analyzed for each individual of the three investigated species (Table 1).

### 4.3. Genome Sequencing and Assembly

Total genomic DNAs of the three *Iris* species were extracted using the modified cetyltrimethylammonium bromide method [89]. Three genomic libraries were prepared using the TruSeq DNA Nano Kit (Illumina, San Diego, CA, USA) and sequenced using NextSeq500 platform (Illumina). A total of 3.2–3.4 gigabases (Gb) of paired-end reads (2 × 150 base pairs [bp]) were generated. Trimmed paired-end reads (Phred score ≥ 20) were assembled using the CLC genome assembler (version 4.06 beta; CLC Inc., Rarhus, Denmark) with the default parameters. SOAP de novo gap closer was used to fill in gaps based on alignments of paired-end reads [90]. Contigs were queried against the non-redundant database of the National Center for Biotechnology Information (NCBI) to identify those representing cp genomes, which were retrieved from the total contigs using Nucmer [91]. The aligned contigs were ordered using the cp genome sequences of *I. koreana* (NC_056174), *I. minutoaurea* (NC_056177), and *I. odaesanensis* (NC_056178) as references [12]. Finally, the trimmed paired-end reads were assembled into complete cp genome sequences using BWA software version 0.7.25 [92] (Figure S1). The newly sequenced chloroplast genomes in the present study were deposited in the NCBI GenBank database.

### 4.4. Genome Annotation and Comparative Analysis

Annotations of *I. koreana*, *I. minutoaurea*, and *I. odaesanensis* cp genomes were performed using GeSeq [93]. Protein-coding sequences were manually curated and verified using Artemis [94] and checked for the quality against the NCBI protein database. The tRNA genes were verified using tRNAscan-SE software version 1.21 [95]. IR-region sequences were verified using IR finder and RepEx [96]. Circular maps of the three *Iris* cp genomes were created using OGDRAW [97]. GC contents of the three cp genomes were analyzed using MEGA7 software [98]. The mVISTA program [99] in Shuffle-LAGAN mode was used for comparative analyses of the cp genomes, with the *I. koreana* cp genome serving as a reference. DnaSP software version 6 [100] was used to calculate the nucleotide diversity (Pi) of cp genomes. Substitution rates (Ka and Ks) were estimated using KaKs_Calculator software version 2.0 [101].

### 4.5. Repeat Analysis

REPuter software was used to identify forward and reverse repeats with the following parameters: a minimum length of 20 bp, identity of 90%, and a Hamming distance of 3 [102]. SSRs were detected using MISA [103], with the minimum number of repeat parameters set to 10, 5, 4, 3, 3, and 3 for mono-, di-, tri-, tetra-, penta-, and hexanucleotides, respectively. Tandem repeats of ≥20 bp were identified using Tandem Repeats Finder [104], with the minimum alignment score of 50 and maximum period size of 500, and the identity of repeats was set to ≥90%.

### 4.6. Phylogenetic Analysis

A total of 31 Iridaceae cp genomes, including three newly sequenced chloroplast genomes, were used in this study, such as *I. koreana*, *I. minutoaurea,* and *I. odaesanensis*, as well as 26 cp published genomes of *Iris* species to date, and two *Crocus* genomes (*Crocus cartwrightianus*, NC_041459; *Crocus sativus* NC_041460), which were used as the outgroup taxa for phylogenetic analysis. Of these, 28 published cp genome sequences were downloaded from the NCBI GenBank database (Table S5). MAFFT [105] was used to align all cp genomes. All aligned genes were extracted using the Geneious program (https://www.geneious.com) and arranged alphabetically. The alignments were filtered to remove ambiguously aligned regions using Gblocks software version 5 [106]. The best-fitting model of nucleotide substitutions was determined using the Akaike information criterion in jModelTest software (version 2.1.10) [107] (Table S6), and the GTR + I + G model was selected. ML analysis was performed using RaxML software version 8.0.5 [108] with 1000 bootstrap replicates. BI analysis was carried out using MrBayes software version 3.2.2 [109], with two independent runs and four chains using Markov chain Monte Carlo simulations, simultaneously running for 5,000,000 generations. Trees were sampled every 5000 generations, with the first 25% discarded as burn-in. The 50% majority-rule consensus trees were used for the estimation of PPs. The reconstructed trees were visualized using FigTree software version 1.4.2 [110].

## 5. Conclusions

Allopolyploidy is an important process contributing to diversification and speciation in angiosperms. This study provides new evidence to further support the allotetraploid origin of the endemic species *Iris koreana.* Patterns of the number and localization of 5S and 35S rDNA loci, along with the chromosome number and genome size, and in-depth analyses of cp genomes of the three taxa, provided evidence supporting the previous hypothesis on the origin of the tetraploid *I. koreana* by hybridization between two diploids *I. minutoaurea* and *I. odaesanensis*. Based on the phylogenetic analysis, together with other evidence, such as molecular cytogenetic (rDNA loci number and localization), the chromosome number, and genome size variation, *I. minutoaurea* is likely to be a putative donor of the maternal genome; however, further analyses involving species-specific molecular cytogenetic markers and GISH are required to interpret the origin of chromosomal variations in *Iris* series *Chinenses*. The results in this study contribute towards the chloroplast genome and molecular cytogenetic evolution of the genus *Iris*.

## Figures and Tables

**Table 1:** Investigated species of *Iris* series *Chinenses* with collection numbers, 5S and 18S rDNA signal numbers and localization, chromosome numbers and genome size estimates.

Species (Collection Number) *	(2*n*) *	5S and 18S rDNA Loci Number (Localization)	Genome Size (pg/1C) *
*Iris koreana* (sck00043)	50	4 (pericentric) and 4 (subterminal)	7.35
*I. minutoaurea* (JC041913)	22	2 (pericentric) and 2 (subterminal)	3.71
*I. odaesanensis* (BKC928)	28	2 (pericentric) and 2 (subterminal)	3.68

* Sourced from Choi et al. [8].

**Table 2:** Characteristics of chloroplast genomes of the three *Iris* species in Korea.

Characteristics/Species	*I. koreana*	*I. minutoaurea*	*I. odaesanensis*
Total cp genome size (bp)	152,259	151,342	155,163
Large single copy (LSC) region (bp)	82,181	81,900	83,879
Inverted repeat (IR) region (bp)	25,799	25,542	26,281
Small single copy (SSC) region (bp)	18,480	18,358	18,722
Total number of genes (unique)	114	114	114
Protein-coding gene (unique)	79	79	79
rRNA (unique)	4	4	4
tRNA (unique)	31	31	31
GC content in total (%)	37.8	37.8	37.8
GC content of LSC (%)	36.0	36.0	36.0
GC content of IR (%)	43.1	43.1	43.2
GC content of SSC (%)	31.2	31.2	31.1

## Data Availability

The data presented in this study will be available on request from the corresponding author.

## Supplementary Materials

The following supporting information can be downloaded at: www.mdpi.com/article/10.3390/ijms231810929/s1.
