# Supplementary material for: Comparative Analyses of Complete Chloroplast Genomes and Karyotypes of Allotetraploid Iris koreana and Its Putative Diploid Parental Species (Iris Series Chinenses, Iridaceae)
Source: Int J Mol Sci. 2022 Sep 18;23(18):10929. doi: 10.3390/ijms231810929 (PMC9504294; doi:10.3390/ijms231810929)
Supplement: Supplementary file 1 [file ijms-23-10929-s001.zip › ijms-1862451-supplementary.pdf]

Supplementary Materials:

Comparative analyses of complete chloroplast genomes and karyotypes of allotetraploid *Iris koreana* and its putative diploid parental species (*Iris* series *Chinenses*, Iridaceae)

Inkyu Park [1,2,†], Bokyung Choi [1†], Hanna Weiss-Schneeweiss [3], Soonku So [4], Hyeon-Ho Myeong [5] and Tae-Soo Jang [1,*]

[1] Department of Biological Science, College of Bioscience and Biotechnology, Chungnam National University, Daejeon 34134, Korea

[2] Department of Biology and Chemistry, Changwon National University, Changwon 51140, Korea;

[3] Department of Botany and Biodiversity Research, University of Vienna, Rennweg 14, A-1030 Vienna, Austria

[4] Plant Conservation Center, Korea National Park Research Institute, 2 Baengnyeonsa-gil, Seolcheon-Myeon, Muju-gun 55557, Jeollabuk-do, Korea

[5] Korea National Park Research Institute, 171 Dangu-ro, Wonju-si 26441, Gangwon-do, Korea

* Correspondence: jangts@cnu.ac.kr; Tel.: +82-42-821-6555

† These authors contributed equally to this work.

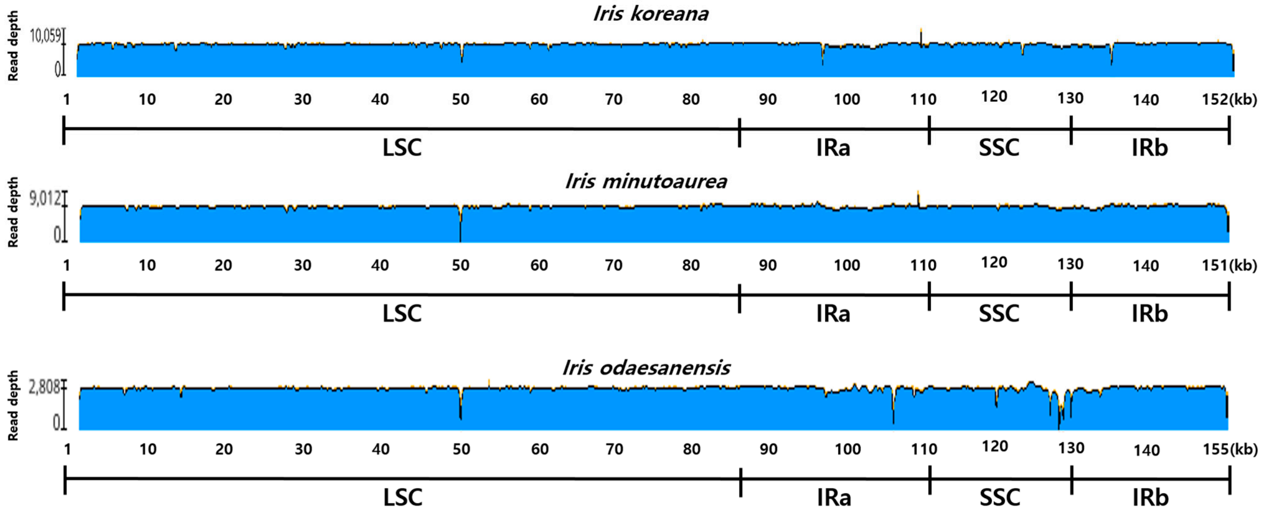

Figure S1. Distribution of paired-end reads mapped onto complete chloroplast genomes of the three *Iris* species. LSC, large single copy region; SSC, small single copy region; IRa, inverted repeat a; IRb, inverted repeat b.

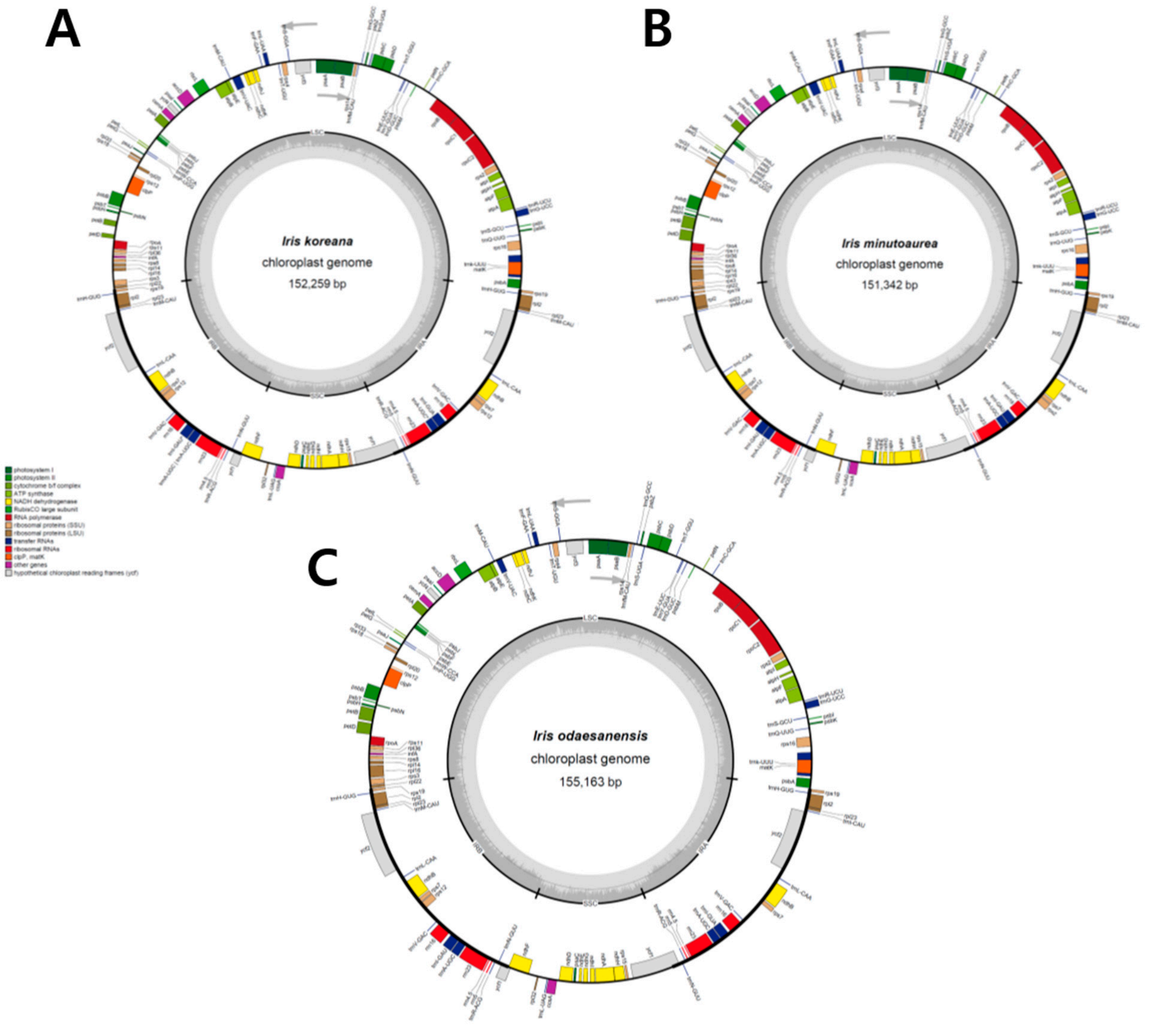

Figure S2. Circular gene map of chloroplast genomes from *Iris*. (A) *I. koreana,* (B) *I. minutoaurea,* and (C) *I. odaesanensis*.

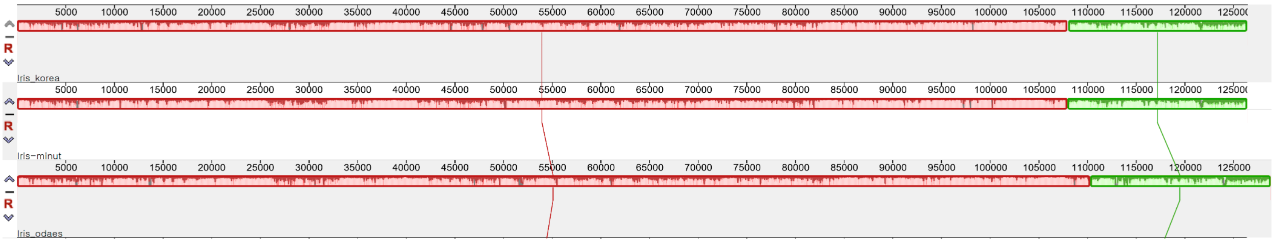

Figure S3. Comparison of complete cp genomes of three *Iris* using the MAUVE algorithm.

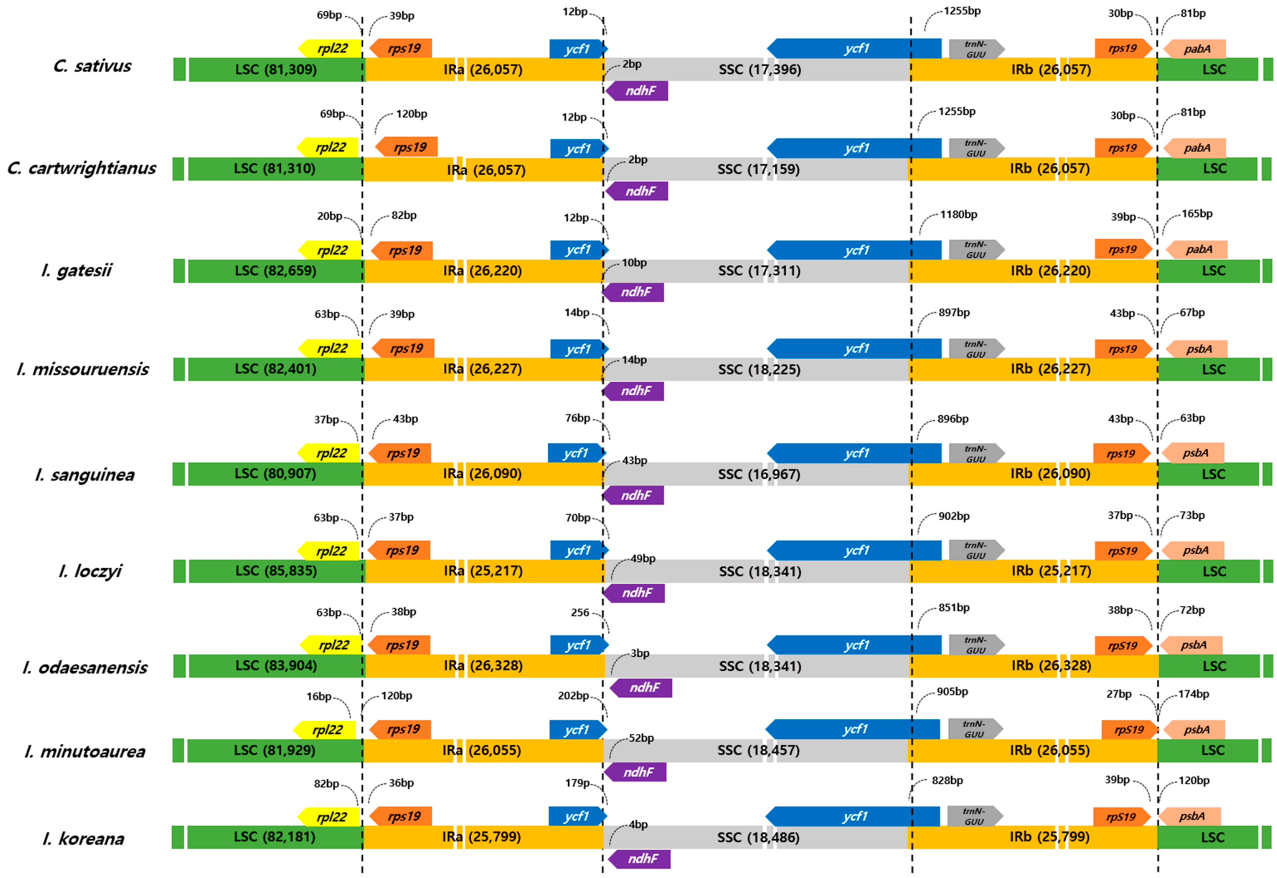

Figure S4. Comparison of the LSC, IR, and SSC junction positions among *Iris* and *Crocus* chloroplast genomes.

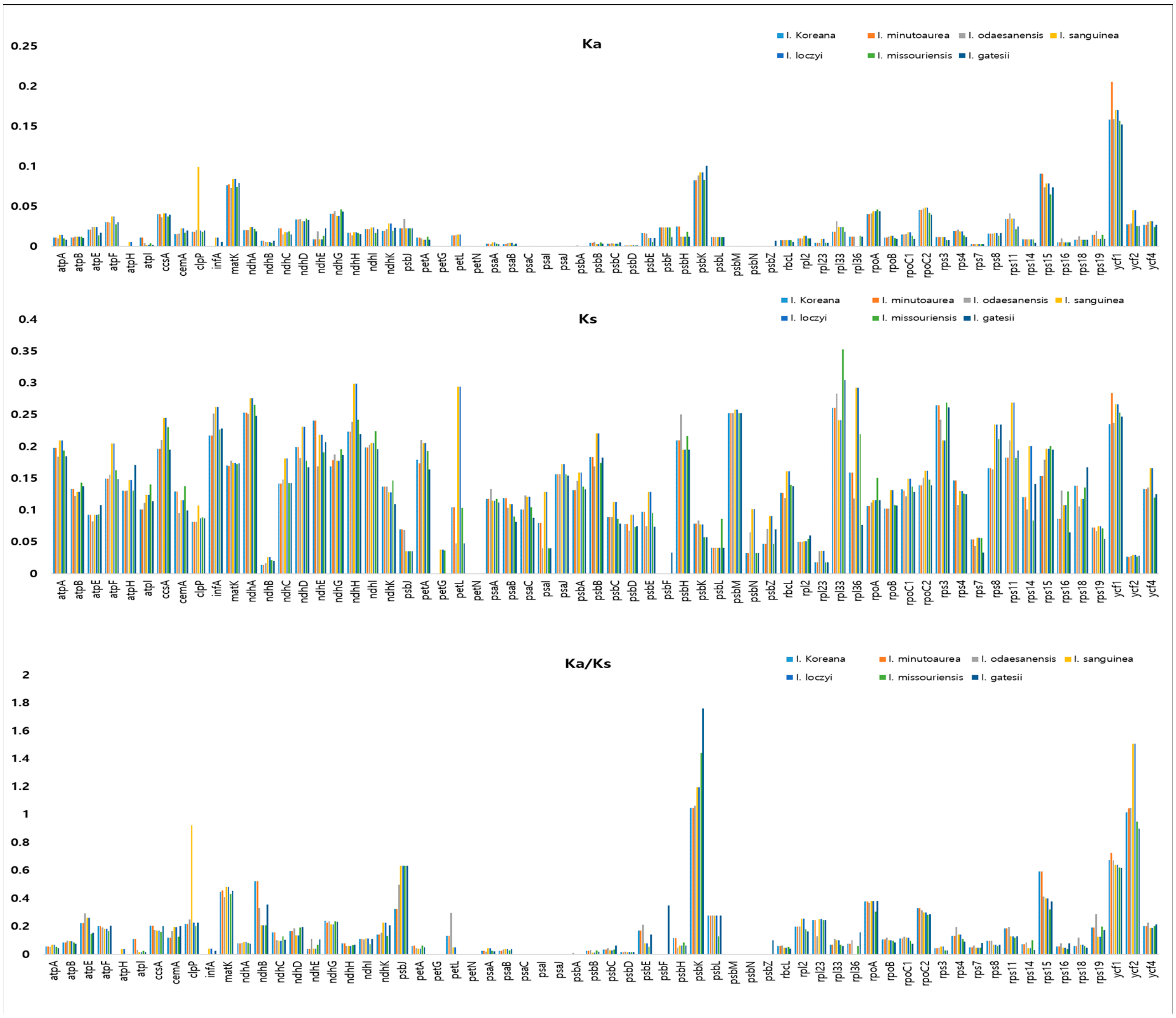

Figure S5. Ka and Ks values for *Iris* chloroplast genomes. The ratios of non-synonymous substitution (Ka) to synonymous substitution (Ks) were calculated for 78 conserved protein-coding sequences using *Crocus sativus* as a reference. Genes with Ka/Ks = 0 are not shown.

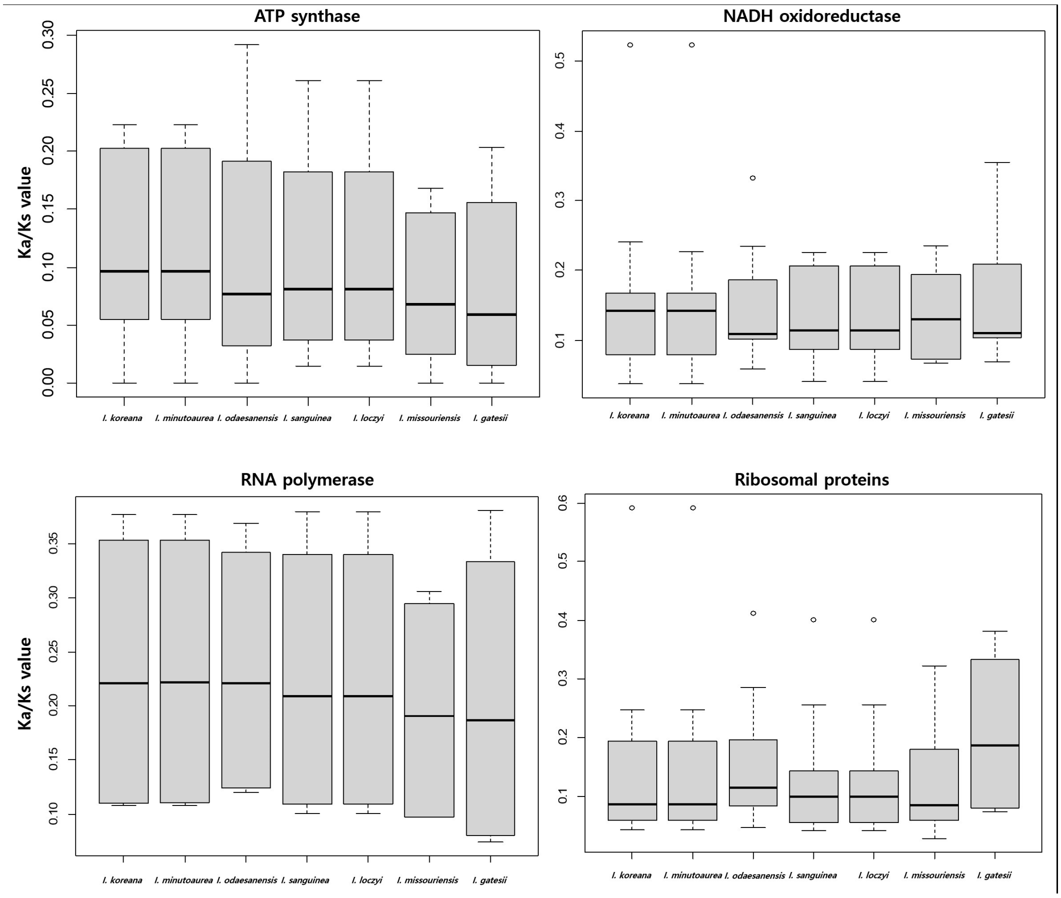

Figure S6. Ka/Ks values for ATP synthase, NADH oxidoreductase, RNA polymerase, and ribosomal proteins related genes in *Iris* chloroplast genomes.

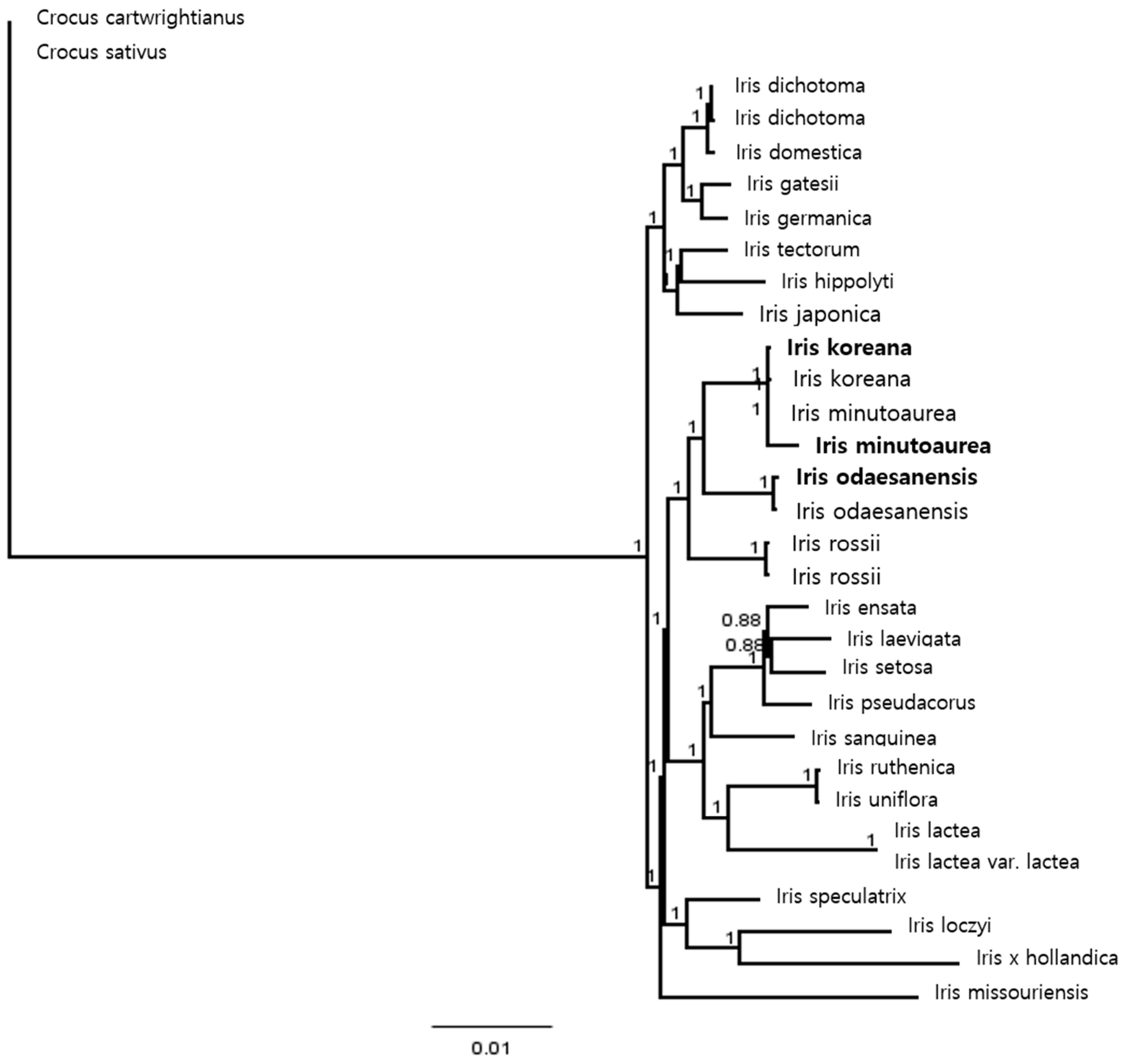

Figure S7. Phylogenetic tree based on 79 CDS from *Iris* using Bayesian posterior probabilities (PP). PP topology is shown with Bayesian posterior probabilities given at each node.

Table S1. Raw and trimmed read data of chloroplast genome of three *Iris* in Korea.

Scientific name	Input reads	Trimmed reads		Total raw bases	Trimmed bases	
I. koreana	11,457,498	10,925,792	95.4%	3,448,706,898	2,880,177,520	83.5%
I. minutoaurea	11,175,776	10,477,480	93.8%	3,363,908,576	2,777,882,232	82.6%
I. odaesanensis	10,839,680	10,301,368	95.0%	3,262,743,680	2,968,585,636	91.0%

Table S2. Genome assembly information for three *Iris* chloroplast genomes.

Scientific name	Aligned reads (#)	Coverage (x)	Cp genome length (bp)
I. koreana	273,699	502	152,297
I. minutoaurea	313,210	556	151,342
I. odaesanensis	385,951	687	155,163

Table S3. Genes in the chloroplast genomes of *Iris* species.

Group of genes	Name of genes
Photosystem I	*psaA, B, C, I, J, ycf3*[2)], *ycf4*
Photosystem II	*psbA, B, C, D, E, F, H, I, J, K, L, M, N, T, Z*
Cytochrome b6/f	*petA, B*[1)], *D*[1)], *G, L, N*
ATP synthase	*atpA, B, E, F*[1)], *H, I*
Rubisco	*rbcL*
NADH oxidoreductase	*ndhA*[1)], *B*[1) 3)], *C, D, E, F, G, H, I, J, K*
Large subunit ribosomal proteins	*rpl2*[1) 3)], *14, 16*[1)], *20, 22, 23*[3)], *32, 33, 36*
Small subunit ribosomal proteins	*rps2, 3, 4, 7*[3)], *8, 11, 12*[2) 3) 4)], *14, 15, 16*[1)], *18, 19*
RNA polymerase	*rpoA, B, C1*[1)], *C2*
Unknown function protein coding gene	*ycf1*[3)], *2*[3)]
Other genes	*accD, ccsA, cemA, clpP*[2)], *matK, infA*
Ribosomal RNAs	*rrn16*[3)], *23*[3)], *4.5*[3)], *5*[3)]
Transfer RNAs	*trnA-UGC*[1)3)], *trnC-GCA, trnD-GUC, trnE-UUC, trnF-GAA, trnfM-CAU, trnG-GCC, trnG-UCC, trnH-GUG, trnI-CAU*[3)], *trnI-GAU*[1),3)], *trnK-UUU, trnL-CAA*[3)], *trnI-GUA, trnL-UAA, trnL-UAG, trnM-CAU, trnN-GUU*[3)], *trnP-UGG, trnQ-UUG, trnR-ACG*[3)], *trnR-UCU, trnS-GCU, trnS-GGA, trnS-UGA, trnT-GGU, trnT-UGU, trnV-GAC*[3)], *trnV-UAC, trnW-CCA, trnY-GUA*

1) Gene containing a single intron, 2) gene containing two introns, 3) two gene copies in IRs, 4) trans-splicing gene.

Table S4. Genic introns in *Iris koreana, I. minutoaurea,* and *I. odaesanensis* chloroplast genomes.

	Gene	Region	exon I	intron I	exon II	intron II	exon III
1	trnk-UUU	LSC	37	2606,2606,2628	35		
2	rps16	LSC	40	853,853,853	212		
3	trnG-UCC	LSC	23	675,675,672	48		
4	atpF	LSC	144	765,766,771	411		
5	rpoC1	LSC	432	731,731,718	1617		
6	ycf3	LSC	128	718,718,712	227	728,728,716	155
7	trnL-UAA	LSC	35	393,393,400	50		
8	trnV-UAC	LSC	39	601,600,605	37		
9	rps12	LSC	114		229		26
10	clpP	LSC	71	789,786,775	292	666,665,667	252
11	petB	LSC	8	748, 740, 747	640		
12	petD	LSC	8	733,733,735	485		
13	rpl16	LSC	9	901,939,944	403		
14	rpl2	LSC	393	664,611,611	432		
15	ndhB	IR	777	699,699,699	756		
16	trnI-GAU	IR	42	941,941,941	35		
17	trnA-UGC	IR	38	808,808,809	35		
18	ndhA	SSC	552	1007,1007,1060	540		

Table S5. Chloroplast genomes from NCBI used for phylogenetic analysis.

No.	Family	Taxon	GenBank accession number
1	Iridaceae	*Iris rossii*	MK593166
2		*Iris setosa*	MK593168
3		*Iris loczyi*	MT254070
4		*Iris lactea* var. *lactea*	MT740331
5		*Iris gatesii*	NC_024936
6		*Iris sanguinea*	NC_029227
7		*Iris missouriensis*	NC_042827
8		*Iris domestica*	NC_050833
9		*Iris tectorum*	NC_056093
10		*Iris dichotoma*	NC_056172
11		*Iris ensata*	NC_056173
12		*Iris koreana*	NC_056174
13		*Iris lactea*	NC_056175
14		*Iris laevigata*	NC_056176
15		*Iris minutoaurea*	NC_056177
16		*Iris odaesanensis*	NC_056178
17		*Iris pseudacorus*	NC_056179
18		*Iris rossii*	NC_056180
19		*Iris ruthenica*	NC_056181
20		*Iris uniflora*	NC_056183
21		*Iris japonica*	NC_060499
22		*Iris germanica*	NC_062594
23		*Iris* x *hollandica*	NC_062595
24		*Iris hippolyti*	OK138594
25		*Iris speculatrix*	OK274247
26		*Iris dichotoma*	OK448492
27	Outgroup	*Crocus cartwrightianus*	NC_041459
28		*Crocus sativus*	NC_041460

Table S6. Selection of best-fitting substitution models selection in CDS sets.

Model	#Param	BIC	AICc	lnL	Invariant	Gamma	R	Freq A	Freq T	Freq C	Freq G
GTR+G+I	69	364086.2	363220.3	-181541	0.524361	0.767056	2.322379	0.309423	0.3089	0.177771	0.203906
GTR+G	68	364522.5	363669.1	-181767	n/a	0.193672	2.230251	0.309423	0.3089	0.177771	0.203906
HKY+G+I	65	365049	364233.2	-182052	0.53173	0.76343	3.295791	0.309423	0.3089	0.177771	0.203906
TN93+G+I	66	365058.6	364230.3	-182049	0.531483	0.762624	3.292712	0.309423	0.3089	0.177771	0.203906
T92+G+I	63	365110.7	364320.1	-182097	0.532494	0.763522	3.293257	0.309162	0.309162	0.190838	0.190838
HKY+G	64	365271.4	364468.2	-182170	n/a	0.189947	2.887204	0.309423	0.3089	0.177771	0.203906
TN93+G	65	365276.8	364461.1	-182166	n/a	0.190307	2.822004	0.309423	0.3089	0.177771	0.203906
T92+G	62	365335.5	364557.4	-182217	n/a	0.189343	2.884985	0.309162	0.309162	0.190838	0.190838
